# Isolation and characteristics of the human rotavirus isolate CY2017

**DOI:** 10.1007/s13337-020-00607-w

**Published:** 2020-06-04

**Authors:** Yan Song, Wei Liu

**Affiliations:** Affiliated Chuiyangliu Hospital of Tsinghua University, Beijing, 100022 China

**Keywords:** Rotavirus, Isolation and identification, Characteristics research

## Abstract

Our study aimed to determine whether a virus from fecal samples is human rotavirus (HRV), the main pathogen that causes autumn diarrhea. Specimens were obtained from the feces from children with autumn diarrhea treated at Chuiyangliu Hospital and used to infect MA104 cells, subcultured and observed by electron microscopy. RNA was extracted, cDNA was synthesized by reverse transcription, and plaque formation and hemagglutination were assessed. The cytopathic effect (CPE) were associated with the fourth passage in subculture. CPE extracts were examined by electron microscopy, which allowed us to observe the shape of HRV particles. Amplification of the VP4 gene from HRV was used to identify the viruses as group A rotavirus. The virus causes red blood cell aggregation. The virus isolate was designated as HRV CY2017.

## Introduction

Diarrhea is common in children. Autumn diarrhea, a subcategory of this disease, is mostly caused by human rotavirus (HRV), which belongs to the genus Rotavirus and the family Reoviridae [7, 13]. Epidemiological investigations have shown that HRV is the main pathogen that causes nonbacterial gastroenteritis, accounting for > 50% of all cases of intestinal viral infection [4, 7]. At present, group A HRV is the world’s most common HRV strain. Indeed, statistics from the World Health Organization indicate that HRV infection can explain approximately 600,000 deaths among children under 5 years globally. Group A HRVs are the most common etiologic agents of severe diarrhea in infants and young children worldwide and are responsible for 2 million hospitalizations globally [13]. In China, HRV infection causes 40–60% of cases of autumn and winter diarrhea in infants and young children [7], and studies have demonstrated that improving sanitation has limited effectiveness in preventing HRV-induced diarrhea [4, 7].

## Material and methods

### Specimen collection

Fecal specimens were obtained from children diagnosed with autumn diarrhea at Chuiyangliu Hospital. The children developed acute inflammatory digestive tract symptoms, such as fever, vomiting, and diarrhea.

### MA104 cells and red blood cells (RBCs)

A transformed Monkey fetal kidney cell line (MA104) was purchased from the China Center for Type Culture Collection of Wuhan University. The cells were subcultured in Dulbecco’s modified Eagle’s medium (DMEM) containing 12% fetal bovine serum. Healthy human RBCs from each of the four human ABO blood groups were obtained from the blood bank at the hospital. RBCs from albino mice, rabbits, and dogs were obtained from sterile animals at the Key Laboratory of Infection and Immunity of the Chinese Academy of Sciences.

### Isolation and testing methods

#### Specimen treatment

Stool specimens were first treated with multiple freeze–thaw cycles without Ca^2+^ or Mg^2+^ in phosphate-buffered saline (PBS, including NaCl 8 g, KCl 0.2 g, Na_2_HPO_4_ 1.44 g, KH_2_PO_4_ 0.24 g, deionized water to 1000 ml) dilution. After centrifugation at 16,099×*g* for 30 m, the supernatant was collected and filtered twice through a 0.22-μm membrane filter and used after a sterility test using an *Escherichia coli* smear.

#### Isolation and culture

First, 30 μg/mL trypsin was added to the specimen, followed by incubation in a water bath at 37 °C for 1 h. A monolayer of MA104 cells with good growth was washed with DMEM. Collected specimens were trypsinized and added to the cell monolayer, followed by the addition of DMEM for culture at 37 °C with 5% CO_2_. Cytopathic effect (CPE)-free cells were subjected to subculture until the CPE was observed. Viruses were collected after the CPE reached 60%.

#### Virus observation under an electron microscope

First, 2000 ml of cell culture was centrifuged at 16,099×*g* at 4 °C. The supernatant was centrifuged at a high speed (268,320×*g*), and the pellet was resuspended in PBS. Observation by electron microscopy was performed after negative staining with phosphotungstic acid [5].

#### RT-PCR for the identification and assay of virus nucleic acids

Freeze-thawed virus cell cultures were centrifuged at 16,099×*g*. dsRNA was obtained after the precipitate was cleaved and extracted, followed by absolute ethanol precipitation [3, 13]. Purified RNAs were examined by reverse transcription–PCR (RT–PCR) using group A rotavirus–VP4 primers [3, 9, 12]. According to the reported complete VP4 nucleotide sequences for group A HRV in GenBank, a pair of primers was designed to amplify the VP4 gene of CY2017 (primers 5′ to 3′, S1CGGGGATCCGGCTATAAAATGGCTTC; S2CGGG TCGACCTCTAGACACTGCTTA). The PCR cycling parameters were as follows: 97 °C for 5 min, followed by 30 cycles of 94 °C for 1 m, 55 °C for 1 m and 72 °C for 2 m, and 72 °C for 10 m. The PCR cycling system (50 μL) consisted of sterilized ultra-pure water 36.5 μL, 10 × PCR Buffer 5 μL, dNTP 4 μL, template 2 μL, primers S1 and S2 (50 pmol/μL) 2 μL, and rTaq enzyme 0.5 μL.

#### Virus plaque assay

Trypsin was added to different dilutions (10^−4^, 10^−5^, and 10^−6^) of virus solutions. After incubation in a water bath at 37 °C for 1 h, the solution was added into a monolayer of MA104 cells with good growth. A control group (without virus) was prepared under the same conditions using a mixture covered with 1% nutrient agarose to observe the monolayer of MA104 cells. Plaque formation was then observed for 5 days [5].

#### Viral hemagglutination and hemagglutination inhibition assays

##### Hemagglutination assay

A CPE-reached cell culture was centrifuged at 16,099×*g*; the supernatant was collected, followed by the dilution of virus samples with PBS to different concentrations for agglutination assays involving four main types of RBCs (human RBCs and RBCs from three different kinds of animals). A total of 10 tubes were used, with nos. 1–9 diluted sequentially and no. 10 as the blank control. Equal amounts of PBS and RBC solution were added and incubated at 37 °C for 1 h [1, 3]. Details are shown in Table 1.

**Table 1:** Hemagglutination assay of the CY2017 isolate

Test tube no.	1	2	3	4	5	6	7	8	9	10
Virus dilution	1:4	1:8	1:16	1:32	1:64	1:128	1:256	1:512	1:1024	–
Volume of virus solution (mL)	0.25	0.25	0.25	0.25	0.25	0.25	0.25	0.25	0.25	–
PBS (mL)	0.25	0.25	0.25	0.25	0.25	0.25	0.25	0.25	0.25	0.50
0.5% RBC (mL)	0.25	0.25	0.25	0.25	0.25	0.25	0.25	0.25	0.25	0.25

##### Hemagglutination inhibition assay

A hemagglutination inhibition assay was then performed on samples in which agglutination occurred or was suspected to occur. A total of 11 tubes were used; nos. 1–9 were serially diluted, no. 10 represented the virus control, and no. 11 was the RBC control. Details are shown in Table 2.

**Table 2:** CY2017 hemagglutination inhibition assay

Test tube no.	1	2	3	4	5	6	7	8	9	10	11
Serum dilution	1:4	1:8	1:16	1:32	1:64	1:128	1:256	1:512	1:1024	–	–
Volume of virus (mL)	0.25	0.25	0.25	0.25	0.25	0.25	0.25	0.25	0.25	–	–
4 units of virus (mL) [12]	0.25	0.25	0.25	0.25	0.25	0.25	0.25	0.25	0.25	0.25	–
0.5% RBC (mL)	0.25	0.25	0.25	0.25	0.25	0.25	0.25	0.25	0.25	0.25	0.25
PBS	–	–	–	–	–	–	–	–	–	0.25	0.50

### Results

#### Viral infection led to the CPE

**Fig. 1:**
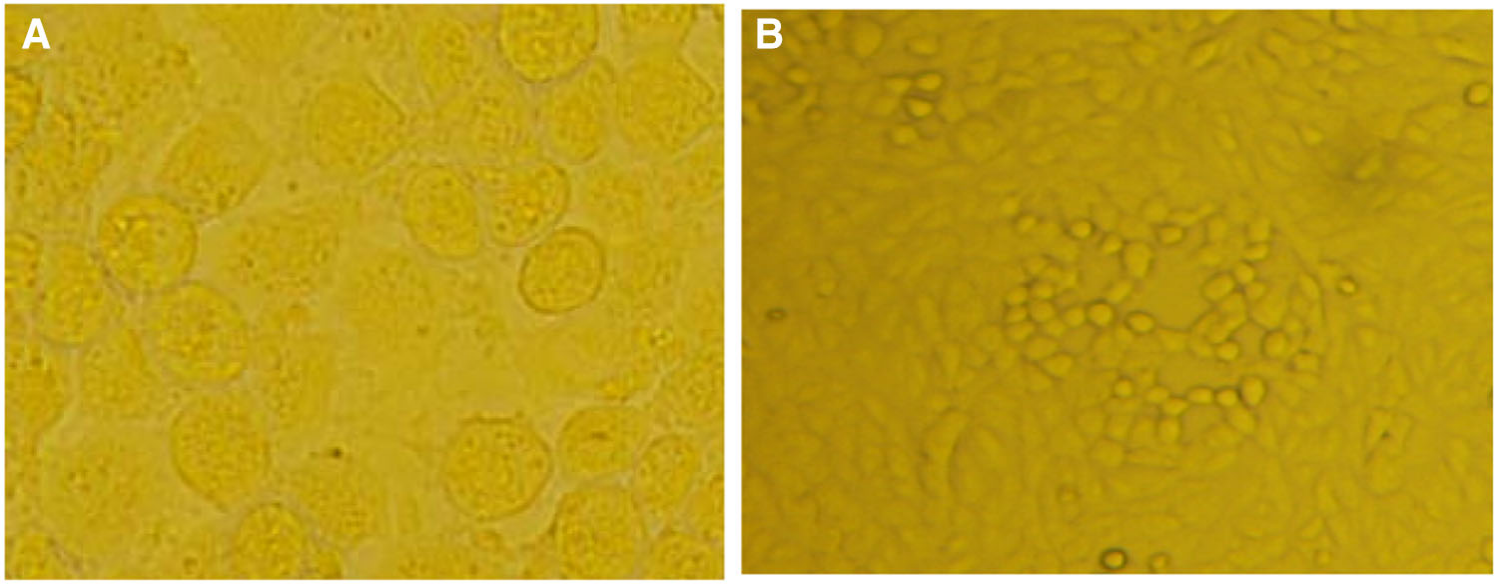
**a** Normal monolayer of MA104 cells (× 150). **b** MA104 CPE caused by CY2017 isolate (× 150)

Virus-infected cells were subcultured, and those at the fourth passage were used for a CPE assay. Obvious effects appeared after 48 h, with cells becoming deformed, detached and dead. The results are shown in Fig. 1b.

#### Virus particles visualized by electron microscopy

**Fig. 2:**
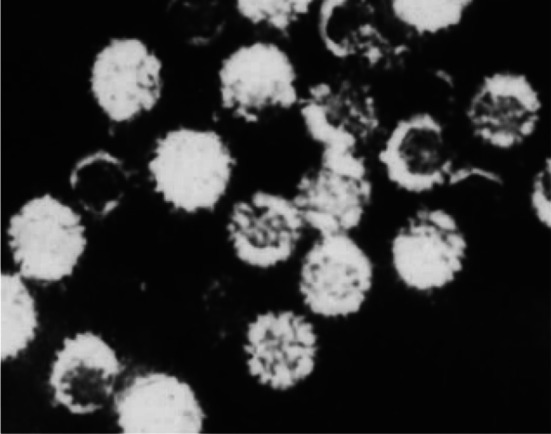
CY2017 virus particle morphology shown under an electron microscope (× 200,000)

Virus particles were circular under an electron microscope, with a diameter of 72–74 nm. They were nonenveloped, the capsid was double layered, and the inner layers of shells were arranged radially, exhibiting a spoke form, a typical feature of rotavirus. A genomic structure was visible in the core, as illustrated in Fig. 2.

#### RT-PCR for the VP4 gene

**Fig. 3:**
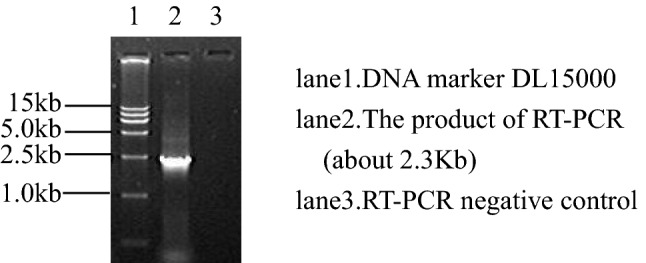
RT-PCR amplification of the VP4 gene from the CY2017 isolate

Total viral RNA was extracted using the SDS-protease K method. RT-PCR of the VP4 main antigen site gene revealed a fragment of approximately 2.3 kb, which was consistent with the expected results (Fig. 3).

#### Virus plaque formation assay

**Fig. 4:**
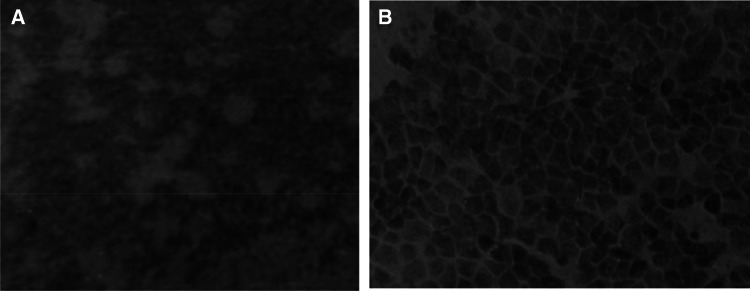
**a** Macroscopic observation of CY2017 virus plaque formation. **b** CY2017 virus plaque (× 150)

The macroscopic background was dotted by colorless plaques. The plaque size was irregular, and more than half of the plaques were approximately 1 mm in diameter. The unstained area represented fragmented, dead, and detached cell masses, and boundaries between adjacent normal cells were defined, as shown in Fig. 4.

#### Virus hemagglutination

Virus strains initially identified as HRV did not aggregate in the majority of RBCs classified according to the human ABO blood group system (type A, B, and AB) or in those from mice, rabbits, or dogs. In contrast, human type O RBCs showed obvious agglutination, with an agglutination titer of 1:128. The agglutination inhibition titer in the human type O hemagglutination inhibition assay was 1:256.

### Discussion

#### Virologic detection and identification of the CY2017 isolate

The key factor in virus isolation involves purification and identification. For virus isolation, we strictly limited specimens to those from relevant cases, as confirmed through epidemiological investigations. Each specimen was cultured independently to ensure only one specimen source. The CPE began to appear as subculture continued into the fourth passage, and the period for lesion appearance gradually decreased as the number of passages increased, indicating a gradual increase in virus infectivity.

Morphological identification was carried out by electron microscopy, which showed virus particles to be mostly circular and occasionally elliptical, with a diameter of 72–74 nm. No capsule was observed, but a double-layered capsid was found; the inner layers of shells were arranged radially, exhibiting a spoke form, a typical feature of HRVs. Members of *Caliciviridae*, such as noroviruses (NVs), which can also cause diarrhea, are morphologically different from HRV. Indeed, NVs are acapsular viruses with a diameter of 26–35 nm, much smaller than HRVs. Regarding culture characteristics, NVs cannot undergo in vitro enrichment culture in cells, another difference from HRVs [10]. The virus isolate in this study was designated HRV isolate CY2017.

According to previous findings, HRVs can be divided into seven groups (A to G) based on viral antigens, with Group A being the most important pathogenetic type [1, 7, 12].

#### Virus plaque formation and blood agglutination assay

Previous research [8] on rotavirus culture in different cell types showed that the use of MA104 cells increased the virus titer most rapidly. In this study, total viral RNA was extracted via the SDS-protease K method. After RNA extraction from the virus culture solution, electrophoresis bands were clearly visible. The CY2017 isolate formed morphologically irregular plaques with different sizes in MA104 cells, a few of which were circular. The plaques initially appeared at 24 h after inoculation, and the proportion reached 25–30% after 48 h and ≥ 80% after 72 h [6, 8].

HRVs are characterized by RBC agglutination. The CY2017 isolate could not agglutinate in most subtypes of RBCs from humans (including types A, B, and AB) or in those from mice, rabbits, or dogs, but it could agglutinate in human type O RBCs [1, 11].

In China, HRV was isolated for the first time in 1985, and many isolates have been obtained. Epidemiological investigations and virus morphological identification and typing have been achieved by employing relatively highly developed technologies [8, 10]. However, poor effects for potential vaccines have been obtained, and research has indicated that improving sanitation is not adequate for reducing HRV-induced autumn diarrhea [1, 2]. Consequently, studies on HRV, especially on effective vaccines and viral invasion-related pathogenesis, are important. The collection of specimens and the isolation and identification of HRV isolate CY2017 described in this paper provide a certain foundation for comparative studies on the characteristics of local isolates, as well as on HRV pathogenicity and host immunity.
